# The NDR Kinase Scaffold HYM1/MO25 Is Essential for MAK2 MAP Kinase Signaling in *Neurospora crassa*

**DOI:** 10.1371/journal.pgen.1002950

**Published:** 2012-09-20

**Authors:** Anne Dettmann, Julia Illgen, Sabine März, Timo Schürg, Andre Fleissner, Stephan Seiler

**Affiliations:** 1 Institute for Microbiology and Genetics, University of Goettingen, Goettingen, Germany; 2 Institute for Genetics, Technische Universität Braunschweig, Braunschweig, Germany; Duke University Medical Center, United States of America

## Abstract

Cell communication is essential for eukaryotic development, but our knowledge of molecules and mechanisms required for intercellular communication is fragmentary. In particular, the connection between signal sensing and regulation of cell polarity is poorly understood. In the filamentous ascomycete *Neurospora crassa*, germinating spores mutually attract each other and subsequently fuse. During these tropic interactions, the two communicating cells rapidly alternate between two different physiological states, probably associated with signal delivery and response. The MAK2 MAP kinase cascade mediates cell–cell signaling. Here, we show that the conserved scaffolding protein HYM1/MO25 controls the cell shape-regulating NDR kinase module as well as the signal-receiving MAP kinase cascade. HYM1 functions as an integral part of the COT1 NDR kinase complex to regulate the interaction with its upstream kinase POD6 and thereby COT1 activity. In addition, HYM1 interacts with NRC1, MEK2, and MAK2, the three kinases of the MAK2 MAP kinase cascade, and co-localizes with MAK2 at the apex of growing cells. During cell fusion, the three kinases of the MAP kinase module as well as HYM1 are recruited to the point of cell–cell contact. *hym-1* mutants phenocopy all defects observed for MAK2 pathway mutants by abolishing MAK2 activity. An NRC1-MEK2 fusion protein reconstitutes MAK2 signaling in *hym-1*, while constitutive activation of NRC1 and MEK2 does not. These data identify HYM1 as a novel regulator of the NRC1-MEK2-MAK2 pathway, which may coordinate NDR and MAP kinase signaling during cell polarity and intercellular communication.

## Author Summary

Intercellular communication and cellular morphogenesis are essential for eukaryotic development. Our knowledge of molecules and mechanisms associated with these processes is, however, fragmentary. In particular, the molecular connection between signal sensing and regulation of cell polarity is poorly understood. Fungal hyphae share with neurons and pollen tubes the distinction of being amongst the most highly polarized cells in biology. The robust genetic tractability of filamentous fungi provides an unparalleled opportunity to determine common principles that underlie polarized growth and its regulation through cell communication. In *Neurospora crassa*, germinating spores mutually attract each other, establish physical contact through polarized tropic growth, and fuse. During this process, the cells rapidly alternate between two different physiological states, probably associated with signal delivery and response. Here, we show that the conserved scaffolding protein HYM1/MO25 interacts with the polarity and cell shape-regulating NDR kinase complex as well as a MAP kinase module, which is essential for cell communication during the tropic interaction. We propose that this dual use of a common regulator in both molecular complexes may represent an intriguing mechanism of linking the perception of external cues with the polarization machinery to coordinate communication and tropic growth of interacting cells.

## Introduction

Appropriate cellular responses to external and internal stimuli depend on the highly orchestrated activity of interconnected signaling cascades. Within these networks, individual proteins can function in more than just one pathway. This raises the question of signal fidelity and the avoidance of undesired crosstalk in response to a specific signal. One crucial level of control arises from the formation of discrete complexes involving scaffold proteins that bind multiple components of a given pathway [1], [2]. By facilitating the transient spatio-temporal organization of the different signaling factors, scaffolds promote kinase-substrate interactions, or kinase activation by upstream components such as membrane bound receptors. Scaffold proteins may also actively participate in signal modulation, for example by recruiting opposing phosphatases to the complex [3]–[7].

The best-studied scaffold is the budding yeast protein Ste5p, which binds the three kinases Ste11p, Ste7p and Fus3p of the mitogen-activated protein kinase (MAP kinase) module of the pheromone response pathway [8], [9]. Upon binding of pheromone to its transmembrane receptor, dissociation of the receptor-associated heterotrimeric G protein triggers the recruitment of Ste5p to the plasma membrane through its interaction with the free Gβγ dimer. This membrane association facilitates the phosphorylation of the MAP kinase kinase kinase (MAPKKK) Ste11p by the membrane-associated p21-activated (PAK) kinase Ste20p and activates the mating pathway. The outcome of this activation includes cell cycle arrest, expression of mating specific genes, reorganization of the cytoskeleton and the control of cell fusion of the two mating partners. Although the mating MAP kinase cascade is highly conserved throughout evolution, the Ste5p scaffold is specific for budding yeast. Even close fungal relatives, such as *Ashbya gossypii* or *Candida albicans* lack obvious homologs.

Recent studies in *Neurospora crassa* indicate that a MAP kinase module homologous to the yeast pheromone response pathway is essential for the communication and subsequent fusion of vegetative cells. The three kinases involved are the MAPKKK NRC1, the MAPKK MEK2 and the MAP kinase MAK2 (homologous to budding yeast Ste11p, Ste7p, and Fus3p, respectively) [10]–[13]. Upstream activators of this pathway, including a postulated secreted signal and its cognate receptor are so far unknown. *N. crassa* and other filamentous fungi possess pheromone receptors, heterotrimeric G-proteins, and PAK kinases [14], [15]. However, these upstream components of the yeast pheromone response pathway are dispensable for vegetative cell communication [16], [17]. Cell-cell signaling and tropic growth of *N. crassa* germlings involves the unusual subcellular dynamics of the MAP kinase MAK2 and SOFT, a conserved, Pezizomycotina-specific protein of unknown molecular function. Both proteins are recruited to the plasma membrane of the tip region of communicating germlings in an oscillating and alternating manner [13]. These observations led to the hypothesis that the two fusion cells coordinately switch between two physiological stages, which are probably related to signal sending and receiving [13], [17], [18].

Genetic data indicate a functional connection between the MAK2 MAP kinase pathway and the nuclear Dbf2-related (NDR) kinase COT1 in *N. crassa* [12]. Mutations in MAK2 pathway components partially suppress *cot-1* defects, such as defective tip elongation and hyperbranching. In addition, MAK2 pathway defects are partially overcome in a *cot-1* background. These data suggest an intricate relationship between COT1 and MAK2 that coordinates polar growth, intercellular communication and cell fusion.

NDR kinases, such as COT1, are central elements for controlling cell polarity, proliferation and development [19], [20]. They associate with proteins of the Mps One binder (MOB) family and are activated by germinal center (GC) kinases of the STE20 super family. A given GC kinase can assemble with various NDR-MOB complexes into distinct signaling modules, which are determined by distinct scaffold proteins [21], [22]. The morphogenetic NDR kinase pathway MOR is defined by the conserved scaffold TAO3/SAX2/FRY in fungi and animals [23]–[26]. In addition, fungal NDR kinases also associate with HYM1, a conserved protein that functions as a second and non-redundant scaffold during MOR-dependent cell polarization [27]–[29]. The homologous animal protein MO25 regulates the activity of the tumor suppressor kinase LKB1, but seems not to be involved in NDR kinase signaling [30], [31]. Recently, it has been shown that MO25 interacts with and regulates the activity of multiple GC kinases, implicating MO25 as a master regulator of this subgroup of STE20-related kinases [32].

To date, *N. crassa* COT1, its co-activators MOB2A and MOB2B, and the activating GC kinase POD6 are among the best-characterized components that regulate polar tip extension and subapical branch formation [33]–[39]. Strains carrying mutations in these genes are viable, but display highly restricted colonial growth with growth-arrested needle-shaped tips and produce massive amounts of extension-arrested new tips along the entire cell. In the present study, we show that *N. crassa* HYM1 functions as scaffold of the COT1-MOB2-POD6 complex to regulate the interaction of the two kinases and thereby COT1 activity. Moreover, HYM1 is essential for activation of the MAK2 MAP kinase pathway. We suggest that this dual use of one scaffold in two kinase pathways may promote the coordination of chemotropic growth and cell-cell communication.

## Results

### HYM1 functions as scaffold protein for the COT1 NDR kinase pathway

**Figure 1:**
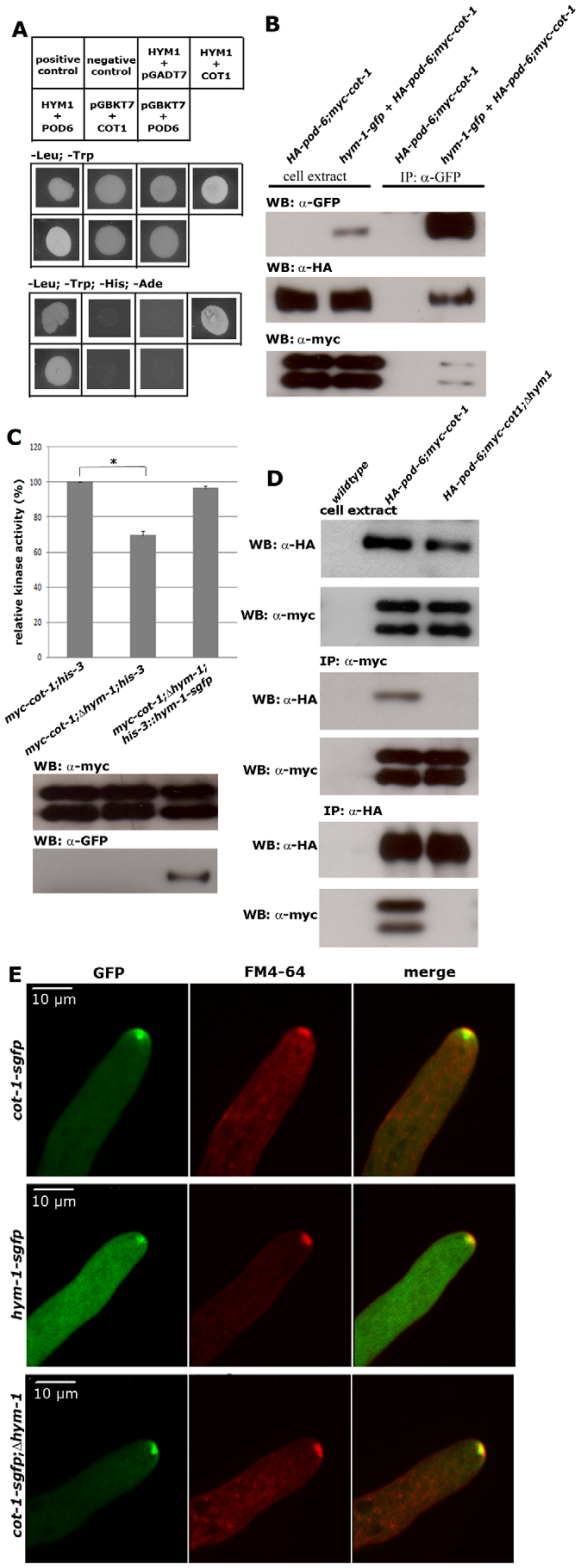
HYM1 functions as scaffold protein for the COT1-POD6 complex. (A) Yeast two hybrid tests identify HYM1 as direct interaction partner of COT1 or POD6. Genes cloned into pGBKT7 and pGADT7 (mentioned as first or second label, respectively) were co-expressed as fusion proteins with the GAL4 DNA-binding domain and activation domains, respectively. Plasmids expressing the indicated proteins either as prey or as bait alone were used as negative controls and pGBKT7-53 (murine p53) and pGADT7-recT (SV40 large T antigen) fusion proteins as positive control. (B) Co-immunoprecipitation of GFP-tagged HYM1 recovered the small and large isoform of myc-COT1 and HA-POD6 from *N. crassa* cell extracts indicating the formation of a COT1-POD6-HYM1 complex *in vivo*. (C) Kinase activity assay of myc-COT1 purified from the indicated strains. Data represent means of five independent experiments (standard deviations are indicated as bars; the star denotes significantly different COT1 activity in the tested strains (p<0,01; unpaired Student's t-test). (D) Reciprocal anti-myc and anti-HA immunoprecipitation experiments from strains co-expressing myc-COT1 and HA-POD6 in a Δ*hym-1* background failed to recover HA-POD6 and myc-COT1, respectively. (E) COT1-GFP accumulated at the hyphal tip as bright spot at the distal side of the *Spitzenkörper* (co-stained with the marker dye FM4-64) and as apex-associated crescent. HYM1, in contrast, did not form this apex-associated crescent and its apical localization fully coincided with the *Spitzenkörper*. Note that both proteins co-localized at constricting septa (Videos S1, S2). COT1-GFP in a Δ*hym-1* background still accumulated as a bright spot at the distal side of the *Spitzenkörper* and as an apex-associated crescent at the hyphal tip.

HYM1 interacts with and regulates morphogenetic NDR kinases in unicellular yeasts, but its function during highly polar filamentous growth is unclear. We identified the uncharacterized ORF NCU03576 as the *N. crassa* homolog of HYM1. The *N. crassa* protein matched HymA of *Aspergillus nidulans* and MO25 of *Schizosaccharomyces pombe* with E-values of 2e^−142^ and 5e^−67^, respectively. To test a potential involvement of HYM1 in the NDR complex we analyzed its interaction with COT1 and POD6 in yeast two-hybrid assays. Both tests rendered positive results (Figure 1A). To corroborate these interactions *in vivo* by immunoprecipitation experiments, we generated a strain that expressed functionally tagged versions of the three proteins. We modified the endogenous loci of *cot-1* and *pod-6* to encode for HA- and myc-tagged kinase genes, respectively, and fused this strain with an isolate that ectopically expressed GFP-tagged HYM1 at the *his-3* locus. The anti-GFP immunoprecipitation recovered both myc-COT1 and HA-POD6 from extracts of *myc-cot-1;HA-pod-6 + hym-1-gfp::his-3*, but not the *myc-cot-1;HA-pod-6* control strain (Figure 1B). In order to test if HYM1 promotes COT1 function, the activity of the kinase was compared in wild type and Δ*hym-1*. COT1 activity purified from Δ*hym-1* was reduced to 70% (SD +/−5; n = 5) of wild type level (Figure 1C). Expression of HYM1-GFP in Δ*hym-1* increased COT1 activity back to wild type level, confirming that the reduced kinase activity was due to the deletion of *hym-1*.

HYM1 might promote COT1 activation by connecting the NDR kinase with its activating kinase POD6. We predicted that in this case the interaction between COT1 and POD6 should be reduced or abolished in a Δ*hym-1* background. To test this hypothesis, we crossed *myc-cot-1;HA-pod-6* with Δ*hym-1* to generate *myc-cot-1;HA-pod-6;*Δ*hym-1*. Reciprocal co-precipitation experiments between myc-COT1 and HA-POD6 revealed that the weak interaction between the two kinases observed in wild type was abolished in a Δ*hym-1* background (Figure 1D). Together, these data indicate that HYM1 functions as a scaffold for COT1 and POD6, thereby promoting COT1 activity.

In order to determine the subcellular localization of COT1 and HYM1, we modified both gene loci to express C-terminally GFP-tagged proteins. The generated strains displayed wild type characteristics, indicating the functionality of the two fusion proteins under the control of their endogenous promoters. COT1-GFP formed an apical membrane-associated crescent in growing hyphal tips and accumulated as a bright, discrete dot that partly overlapped with the distal region of the *Spitzenkörper* (Figure 1E). The *Spitzenkörper* is an apex-associated cluster of vesicles, cytoskeletal elements and polarity factors that serves as vesicle supply center and guides polar tip growth [40]–[42]. HYM1-GFP, in contrast, did not form this apical cap and fully co-localized with the *Spitzenkörper* (Figure 1E). The intensity of the apical localization was higher in COT1-GFP compared to HYM1-GFP, a difference that was also reflected by a ca. 15-fold higher expression level of COT1 in comparison to HYM1 (Figure S1). Moreover, both proteins also labeled constricting septa and accumulated around the septal pore of mature septa (Videos S1, S2). Deletion of *hym-1* did not alter the apical and septum localization of COT1-GFP (Figure 1E), indicating that the scaffold is dispensable for proper localization of the NDR kinase. In summary, these data establish HYM1 as a functional component of the COT1-POD6 kinase complex. However, in contrast to the situation in unicellular yeasts [27], [28], [43], [44], HYM1 is not essential for MOR signaling in *N. crassa*.

### HYM1 is essential for MAK2 MAP kinase activity

**Figure 2:**
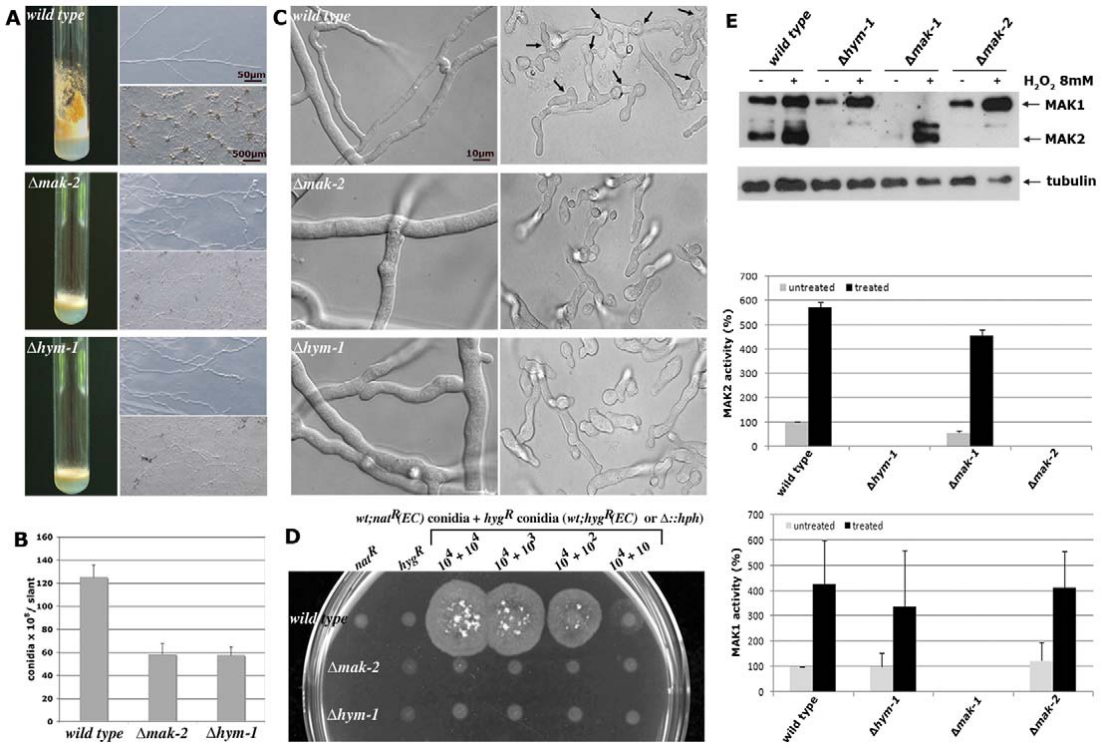
Δ*hym-1* displays phenotypic characteristics of MAK2 MAP kinase pathway mutants. (A) Phenotypic characterization of Δ*hym-1*, Δ*mak-2* and wild type regarding macroscopic morphology and conidiation pattern (left panel: growth in slants for 5 days on minimal medium), hyphal morphology (upper right panel; bar), and protoperithecia formation (lower right panel). (B) Production of conidiospores was quantified by counting conidia generated in slants grown at room temperature for 5 days (n = 5; standard deviations are indicated as bars). (C) HYM1 and MAK2 are required for vegetative cell fusion. Hyphal (left panel) and germling fusion events (right panel; fusion events are indicated by arrows) were assessed by light microscopy in wild type, Δ*hym-1* and Δ*mak-2* cultures. Cell fusion was not observed in Δ*hym-1* and Δ*mak-2*. (D) Quantification of cell fusion competence and formation of forced heterokarya that grew on double-selective media supplemented with 150 µg/ml hygromycin and 20 µg/ml nourseothricin. 10^4^ hygromycin-resistant conidiospores of the indicated strains (“wild type” carried an ectopically integrated (EC) hygromycin-resistance cassette, while the hygromycin-resistance cassette was used to replace the two gene deletions in Δ*hym-1* and Δ*mak-2*) were plated alone (second column) or in decreasing concentrations together with a second “wild type” strain that carried an ectopically integrated nourseothricin-resistance cassette (column 3–6). Column 1 indicated lack of growth of the “wild type” nat^R^ control strain. 1% sorbose was added to Vogels minimal medium to restrict radial growth of the forming colonies generated by forced heterokaryons after incubation for 3 days at room temperature. (E) HYM1 is required for MAK2 activity. Total soluble protein was extracted from the indicated strains grown in the presence or absence of 8 mM H_2_O_2_. Western blot analysis with anti-phospho-p42/p44 antibody detected activated MAK1 and MAK2, respectively. Equal loading was confirmed by re-probing the membrane with anti-tubulin antibody. A typical Western blot is shown. MAK1 and MAK2 activities from 5 independent experiments were quantified for the diagrams.

Mutations in the *cot-1*, *pod-6* or *mob-2a/2b* genes, which define the central components of the *N. crassa* MOR pathway, share characteristic defects, such as the inhibition of polar tip extension and the initiation of extension-arrested new tips along the entire cell [33]–[35], [37], [39]. In contrast, Δ*hym-1* did not display this barbed-wired appearance, but generated defects that were highly reminiscent of strains deficient in components of the *mak-2* MAP kinase pathway [10], [12], [13], [45]. Both, Δ*hym-1* and Δ*mak-2* mutants were characterized by a reduced hyphal growth rate (ca. 35% of wild type) and highly knobby cell morphology, stunted aerial mycelium formation and de-repressed conidiation. In addition, both mutants were unable to develop female sexual structures, called protoperithecia (Figure 2A, 2B). Moreover, similar to MAK2 pathway mutants, germinating conidia of Δ*hym-1* or hyphal cells within an established Δ*hym-1* colony showed no mutual attraction and self-fusion (Figure 2C, 2D).

A similar cell fusion deficiency is described for strains defective in components of the MAK1 cell wall integrity MAP kinase pathway [12], [46]. However, the relationship between the MAK1 and MAK2 MAP kinase modules during hyphal fusion is unresolved. To test if HYM1 might influence the activity of these signaling modules, we compared the phosphorylation status of the two MAP kinases in wild type and Δ*hym-1* by using phospho-specific antibodies against the activated proteins. In wild type cells, MAK1 and MAK2 displayed basal activities that can be stimulated ca. 5- to 10-fold under conditions of oxidative stress [12], [47]. MAK1 displayed wild type activities in non-stressed and H_2_O_2_-stimulated Δ*hym-1*, but MAK2 activity was completely abolished in Δ*hym*-1 (Figure 2E).

**Figure 3:**
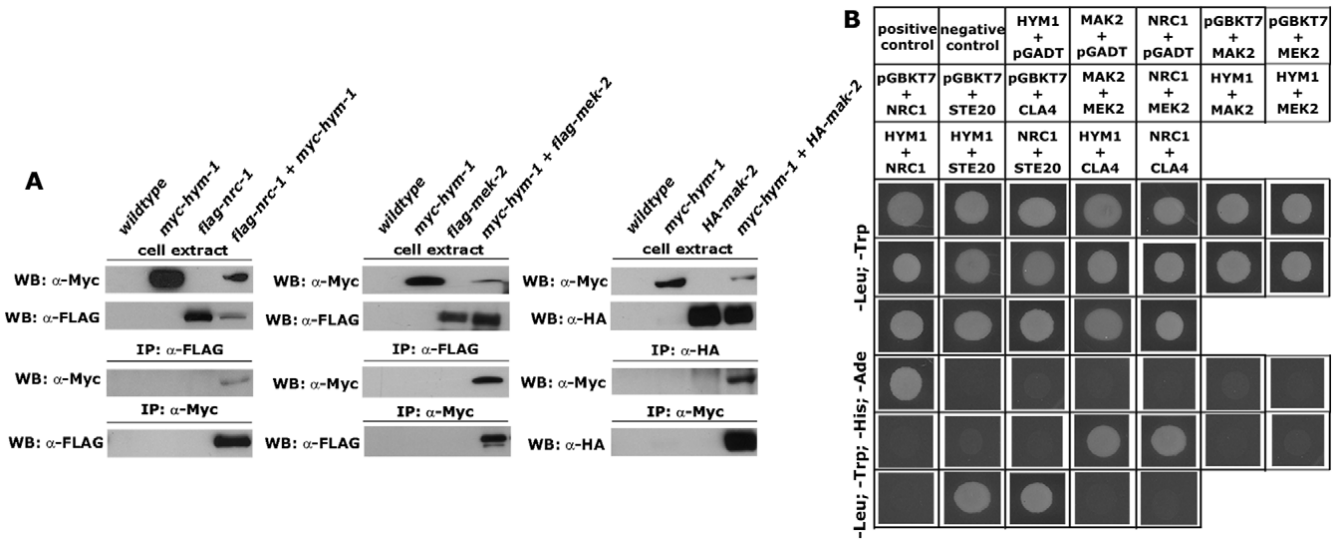
HYM1 interacts with components of the MAK2 MAP kinase pathway. (A) Co-immunoprecipitation experiments of forced heterokaryons expressing myc-tagged HYM1 with flag-NRC1, flag-MEK2 or HA-MAK2. Interactions were observed between HYM1 and all three kinases of the MAK2 pathway. (B) Yeast two-hybrid analysis of HYM1 and the kinases NRC1, MEK2 and MAK2 revealed no physical interactions between the tested protein pairs. Interactions between NRC1 and MEK2 and between MEK2 and MAK2 were used as positive controls. Moreover, HYM1 and NRC1 interacted with the PAK kinase STE20, but not the homologous kinase CLA4.

Driven by these results, we asked if HYM1 interacted with the MAK2 pathway *in vivo* by performing co-precipitation experiments. We detected weak, but consistent interactions between myc-HYM1 and flag- and HA-tagged versions of three kinases of the MAK2 module (Figure 3A). However, the interaction between HA-MAK2 and its upstream kinase flag-MEK2, which was used as positive control, was stronger and stable after several washes with 300 mM NaCl, while the interactions between HYM1 and the three kinases were only detected in immunoprecipitations lacking the high salt washing steps (Figure S2). Moreover, yeast two-hybrid tests between HYM1 and the three kinases of the MAK2 MAP kinase cascade were negative (Figure 3B). In control experiments, we observed interactions between NRC1 and MEK2 and between MEK2 and MAK2, confirming the functionality of the yeast two-hybrid constructs and the physical interaction of the NRC1-MEK2-MAK2 cascade. In summary, HYM1 is essential for MAK2 activity, but appears to interact with the three MAP kinases only in an indirect manner, probably as part of a larger protein complex. In line with this hypothesis, we found that the PAK kinase STE20/NCU03894 physically interacted with HYM1 and with NRC1 in yeast two-hybrid assays. The second PAK kinase present in *N. crassa*, CLA4/NCU00406, which we used as control, did not interact with HYM1 or NRC1 (Figure 3B).

### HYM1 is required for signal transduction through the NRC1-MEK2-MAK2 kinase cascade

**Figure 4:**
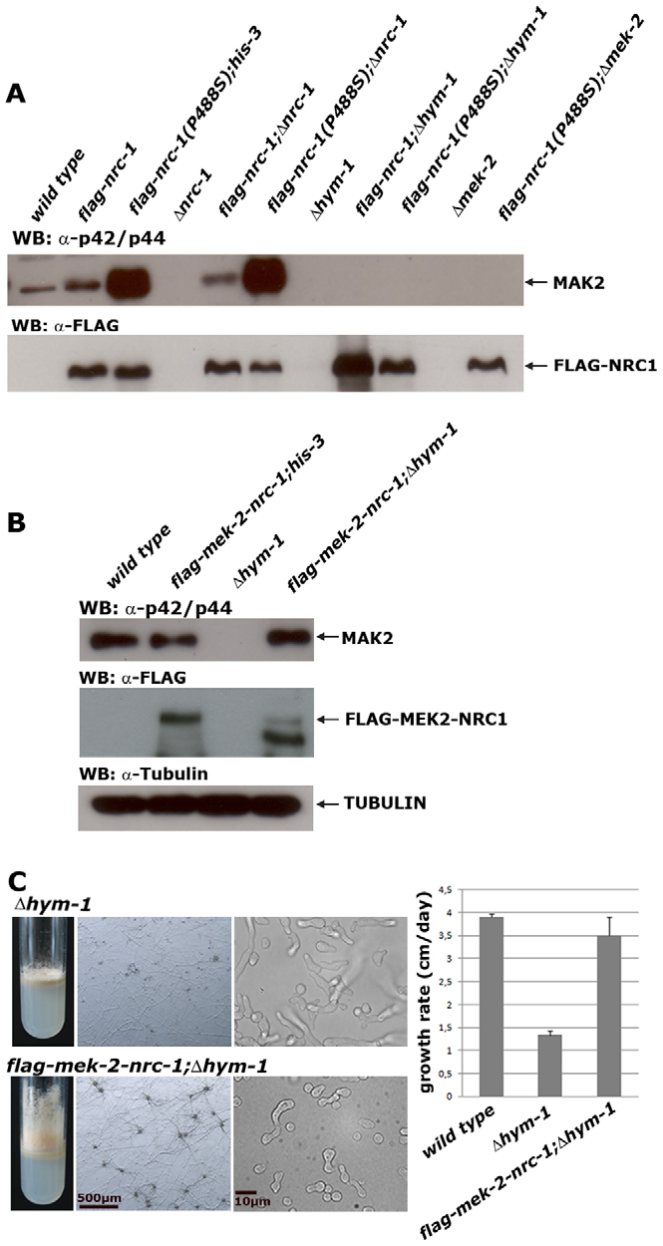
HYM1 is required for signal transduction through the entire MAK2 kinase cascade. (A) Constitutive hyperactive versions of NRC1 (NRC1(P448S)) and MEK2 (MEK2(S212D;T216D) do not activate MAK2 in a Δ*hym-1* background. Total soluble protein was extracted from the indicated strains grown at 25°C and Western blot analyzed with anti-phospho-p42/p44 antibody to probe for MAK2 activity. Equal loading was confirmed by re-probing the membrane with anti-FLAG antibody. (B) Expression of a MEK2-NRC1 fusion protein in Δ*hym-1* resulted in wild type levels of active MAK2 (determined by Western blot experiments with anti-phospho-p42/p44 antibody). Note that the stability of the MEK2-NRC1 fusion protein seems reduced in Δ*hym-1*, resulting in the consistent appearance of potential degradation products with smaller size (B). An anti-tubulin blot served as loading control to indicate equal protein levels. (C) Expression of a MEK2-NRC1 fusion protein complemented all Δ*hym-1* defects (panel 1: aerial mycelium formation in slants; panel 2: sexual development/protoperithecia formation; panel 3: germling fusion; panel 4: growth rate).

Based on these results, HYM1 could function upstream of the MAK2 pathway or as a component of a scaffold complex that connects the three kinases of the MAP kinase cascade. To distinguish between these two possibilities we employed constitutive activated components of the MAK2 MAP kinase module. We hypothesized that this activation would complement Δ*hym-1* defects in the first case, while the activation should not be transferred to the downstream components in the latter one. First, we constructed a proline 448 to serine-substituted version of NRC1. Homologous mutations have previously been shown to confer constitutive activity of related fungal MAPKKKs [48], [49]. Ectopic expression of flag-NRC1(P448S) in the *N. crassa* wild type and Δ*nrc-1* control strains increased MAK2 activity ca. 12–fold (Figure 4A; Table 1). When we expressed this construct in Δ*mek-2* as control, hyperactivation of MAK2 was blocked, confirming that the constitutive NRC1 signal is transmitted through the MAPK cascade. No MAK2 activity was detected when we expressed flag-NRC1(P448S) in Δ*hym-1*. Analogous results were obtained, when we expressed a constitutive active version of MEK2 in these strains. Expression of MEK2(S212D;T216D) resulted in MAK2 hyperactivation in wild type and Δ*mek-2*, but not in Δ*hym-1*. Thus, HYM1 is required for signal transmission through the entire NRC1-MEK2-MAK2 MAP kinase cascade (Table 1).

**Table 1:** Phenotypic characteristics of constitutive hyperactive NRC1 and MEK2 variants in the indicated strains.

	*his-3*	*nrc-1::his-3*	*nrc-1(P448S)::his-3*	*mek-2::his-3*	*mek-2(DD)::his-3*
Growth rate*	3,8(±0,2)	3,2(±0,1)	3,3(±0,2)	3,6(±0,2)	3,1(±0,1)
Aerial mycelium	wild type	wild type	reduced	wild type	wild type
Conidiation	wild type	wild type	reduced	wild type	wild type
Protoperithecia	wild type	wild type	delayed	wild type	wild type
Germling fusion	wild type	wild type	wild type	wild type	wild type
MAK2 activity**	100%	120% (±15%)	1260% (±35%)	125% (±15%)	1120% (±30%)

	Δ*nrc-1;his-3*	Δ*nrc-1;nrc-1::his-3*	Δ*nrc-1;nrc-1(P448S)::his-3*	/	/
Growth rate	1,6(±0,2)	3,3(±0,1)	3,1(±0,2)	/	/
Aerial mycelium	no	wild type	reduced	/	/
Conidiation	derepressed	wild type	reduced	/	/
Protoperithecia	no	wild type	very few	/	/
Germling fusion	no	wild type	no	/	/
MAK2 activity	0%	115% (±25%)	1410% (±25%)	/	/

	Δ*hym-1;his-3*	Δ*hym-1;nrc-1::his-3*	Δ*hym-1;nrc-1(P448S)::his-3*	Δ*hym-1;mek-2::his-3*	Δ*hym-1;mek-2(DD)::his-3*
Growth rate	1,4(±0,1)	1,4(±0,3)	2,9(±0,2)	1,4(±0,3)	2,8(±0,2)
Aerial mycelium	no	no	no	no	no
Conidiation	derepressed	derepressed	derepressed	derepressed	derepressed
Protoperithecia	no	no	very few	no	very few
Germling fusion	no	no	no	no	no
MAK2 activity	0%	0%	0%	0%	0%

	Δ*mek-2;his-3*	/	Δ*mek-2;nrc-1(P448S)::his-3*	Δ*mek-2;mek-2::his-3*	Δ*mek-2;mek-2(DD)::his-3*
Growth rate	1,3(±0,1)	/	1,3(±0,3)	3,1(±0,3)	2,8(±0,4)
Aerial mycelium	no	/	no	wild type	reduced
Conidiation	derepressed	/	derepressed	wild type	reduced
Protoperithecia	no	/	no	wild type	very few
Cell fusion	no	/	no	wild type	no
MAK2 activity	0%	/	0%	130% (±20%)	1090% (±35%)

* in cm/day.

** Basal MAK2 activity was determined as described in Material and Methods; MAK2 activity was calculated relative to wild type, whose activity was set to 100% (n≥3).

Consistent with the observed MAK2 activities in these strains, expression of flag-NRC1(P448S) rescued the vegetative hyphal growth defects of Δ*nrc-1*, but not of Δ*mek-2*, while flag-MEK2(S212D;T216D) complemented Δ*mek-2* (Table 1; Figure S3). Interestingly, the two constitutive hyperactive kinase variants also increased the growth rate of Δ*hym-1*, although we were unable to detect MAK2 activity in this strain. Thus, a basal signal transduction rate below the detection level of the p42/44 anti-ERK antibodies seems sufficient for sustained vegetative growth in the absence of HYM1. In contrast, the cell fusion defects of Δ*nrc-1*, Δ*mek-2* and Δ*hym-1* were not complemented by flag-NRC1(P448S) or MEK2(S212D;T216D), suggesting that cell fusion requires signal based, adjustable MAP kinase activation.

To further support the finding that HYM1 promotes signal transduction through the MAP kinase module, we tested if the artificial tethering of two of the kinases rescued the Δ*hym-1* defects. A flag-MEK2-NRC1 fusion protein was generated and expressed in the different mutants. This construct rescued all defects of Δ*mek-2* and Δ*nrc-1* and resulted in wild type levels of MAK2 activity in both strains, confirming the functionality of the two fused kinases. Moreover, this construct resulted in wild type MAK2 activity levels when expressed in Δ*hym-1* and fully complemented the vegetative and developmental defects of Δ*hym-1* (Figure 4B, 4C). These data indicate that HYM1 functions as part of an essential adaptor complex that connects the components of the NRC1-MEK2-MAK2 cascade to allow signaling through the cell fusion MAP kinase pathway.

### The three kinases of the MAK2 MAP kinase module and HYM1 are recruited to the contact zone of germling fusion pairs

**Figure 5:**
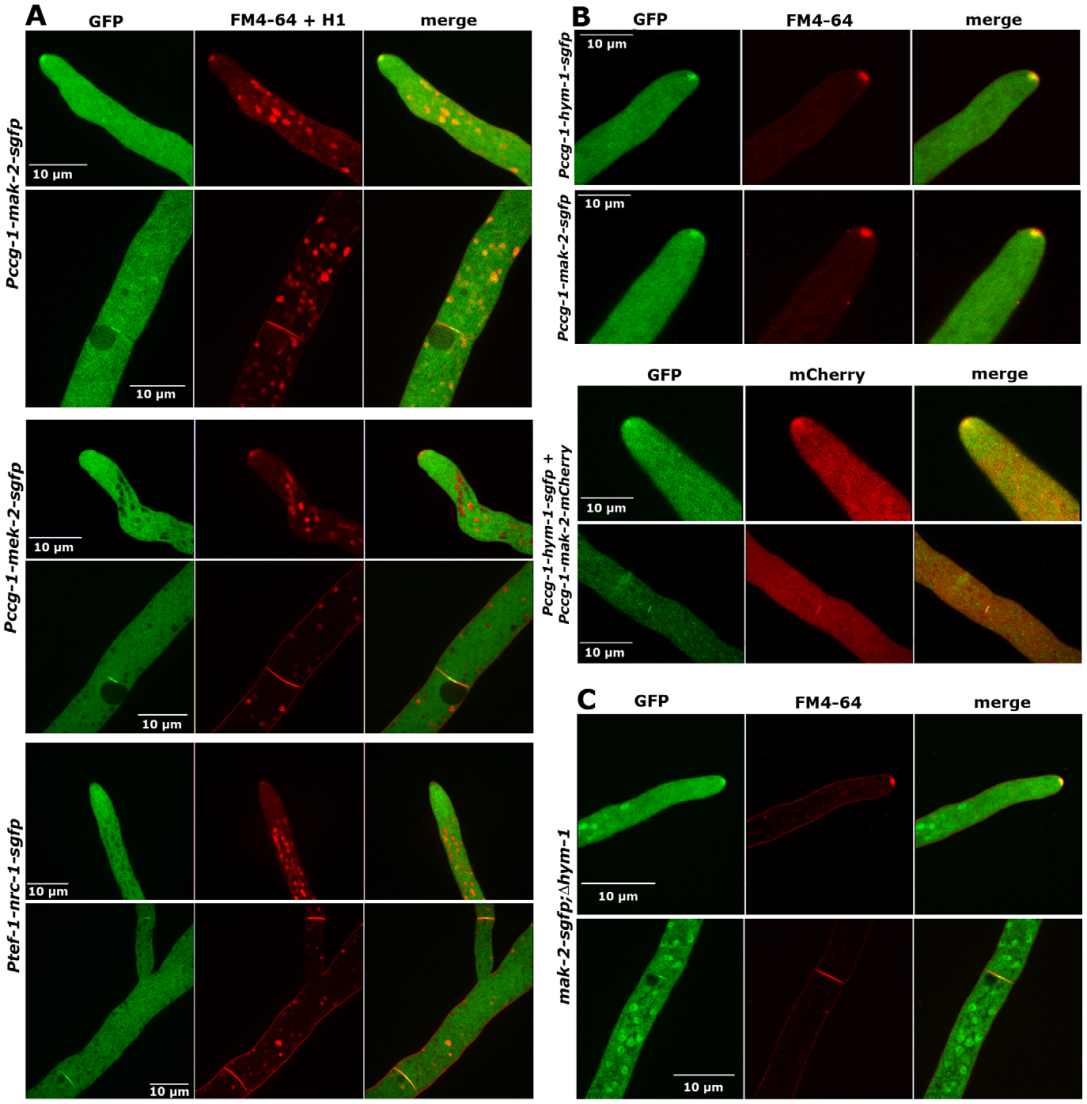
NRC1, MEK2, and MAK2 display different localization patterns in vegetative hyphae. (A) MAK2-GFP accumulated at the hyphal tip and the forming septum, while MEK2 and NRC1 only labeled the septum, but not the cell apex. Note that MAK2 has a cytosolic and nuclear distribution, while MEK2 and NRC1 are exclusive cytosolic proteins. Nuclei, hyphal plasma membrane/septa were co-labeled with histone H1-RFP and FM4-64, respectively, and detected in the red channel. (B) HYM1 and MAK2 co-localized in the *Spitzenkörper* at the hyphal tip and at constricting septa. Upper panel: comparison of hyphal tips of cells expressing HYM1-GFP and MAK2-GFP; lower panel: co-localization of co-expressed HYM1-GFP and MAK2-mCherry. (C) MAK2-GFP accumulated in the nucleus in a Δ*hym-1* background.

The interactions of the three MAK2 pathway kinases and of the kinases with HYM1 suggest a similar subcellular localization of all proteins. We have recently shown that MAK2 is recruited to the tips of two communicating germlings in an oscillatory manner [13]. Its localization in mature hyphae or the subcellular dynamics of the two upstream kinases were unknown. We determined that MAK2-GFP expressed under the control of the *ccg-1* promoter localized in the cytoplasm, was not excluded from the nuclei and accumulated in the *Spitzenkörper* of growing hyphal tips (Figure 5A). Moreover, MAK2 labeled constricting septa and the septal pore of mature septa (Figure 5A; Video S3). Thus, MAK2 displayed a localization pattern highly similar to the localization described for HYM1. Co-localization experiments employing MAK2-mCherry and HYM1-GFP fusion proteins confirmed the co-localization of both factors in the *Spitzenkörper* of growing hyphal tips and at septa (Figure 5B). To exclude that the used promoter and thus the ca. two-fold higher expression level of MAK2 affected its localization, we compared two strains that expressed MAK2-GFP under the control of the native and the *ccg-1* promoter, but no differences in MAK2 localization were observed (Figures S1, S4). Functional MEK2-GFP and NRC1-GFP fusion proteins, expressed under the control of their native and the *ccg-1* promoters, did not display any apical accumulation, but localized in the cytosol and accumulated around mature septal pores (Figure 5A). Moreover, both proteins were associated with constricting septa (Videos S4, S5). In contrast to MAK2, MEK2 and NRC1 were excluded from nuclei (Figure 5A). When we addressed the localization of the three kinases in a *Δhym-1* background, we found that MAK2-GFP, but not the other two kinases, strongly accumulated in the nucleus in addition to the described localization at the hyphal tip and the septum (Figure 5C). These data indicate differential functions of MAK2 and its upstream kinases NRC1 and MEK2 at the tips of vegetatively growing cells and suggest a function of HYM1 in regulating the nuclear versus cytosolic accumulation of MAK2.

**Figure 6:**
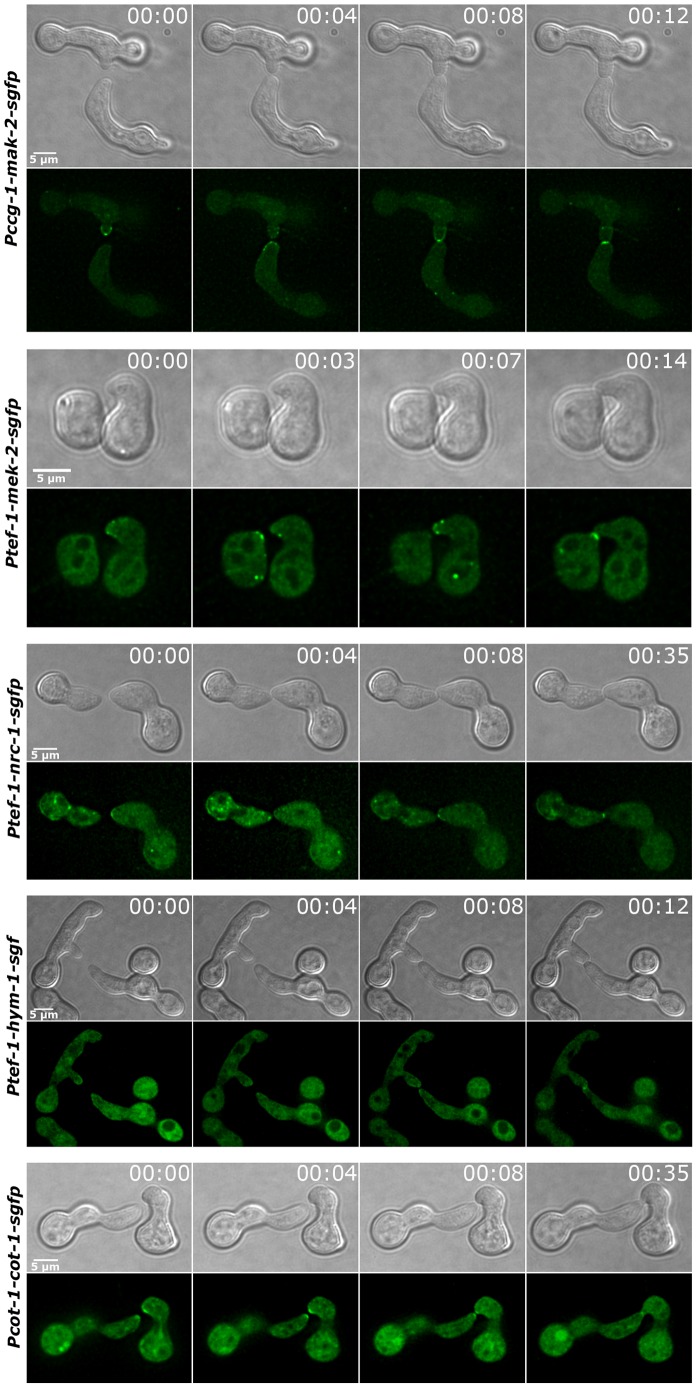
Components of the MAP kinase cascade oscillate during germling fusion. Dynamic localization of MAK2-GFP, MEK2-GFP and NRC1-GFP at opposing tips of interacting germlings. HYM1-GFP is recruited to the contact zone of germling fusion pairs. COT1-GFP associated with both tips during tropic growth of communicating germlings.

**Figure 7:**
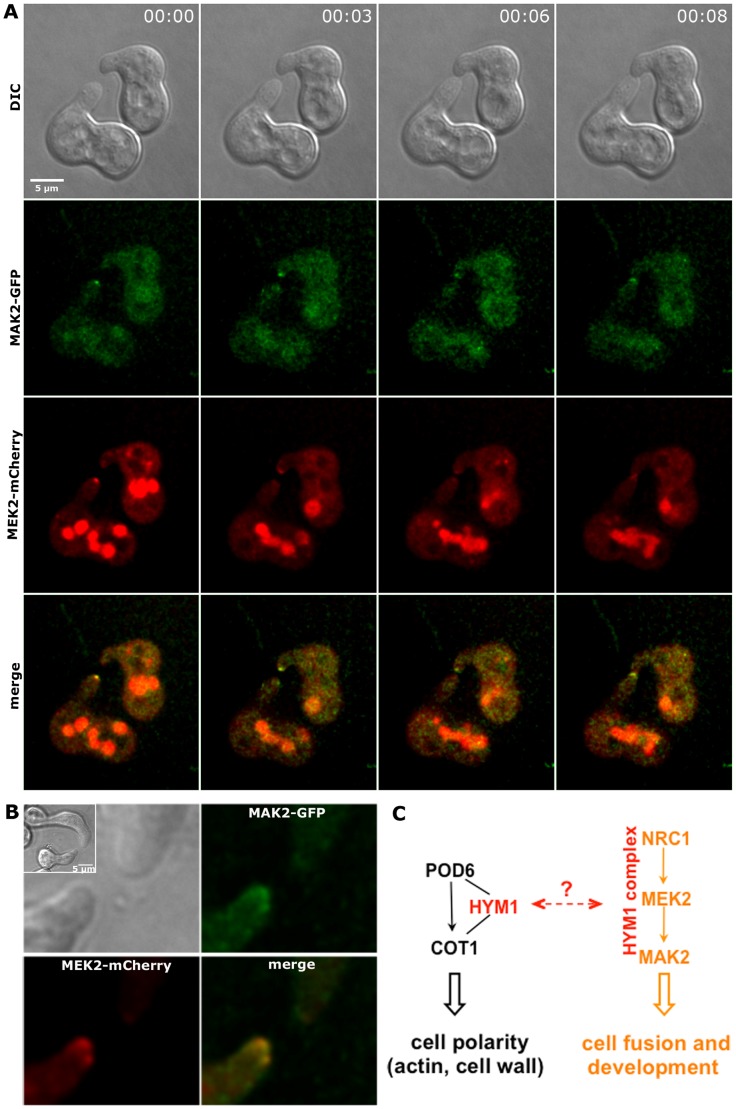
MAK2-GFP and MEK2-mCherry co-localize at the tips of interacting germlings. (A) MAK2-GFP and MEK2-mCherry recruitment to the germling tips oscillated synchronously during tropic growth. (B) MAK2-GFP and MEK2-mCherry co-localized in complexes at the tips of interacting germlings. Images were taken at one time point. DIC Inlay shows the entire germlings. (C) Model for the potential interactions of the COT1 and MAK2 signal pathways through the common scaffold HYM1; see text for details.

Since MEK2, NRC1 and HYM1 are essential for germling fusion, we tested if GFP fusion constructs of these components displayed a dynamic localization to communicating germling tips, as described for MAK2 [13]. In germlings of strains expressing either one the three constructs under their native promoters barely any fluorescence was detectable. Therefore, strains carrying functional *gfp* fusion constructs under the control of the stronger *ccg-1* or *tef-1* promoters were employed. MEK2-GFP showed subcellular dynamics comparable to MAK2 in communicating partner cells (Figure 6). Based on this observation and the physical interaction of the two kinases (Figure 3) we tested if the two proteins co-localized during the dynamic recruitment associated with tropic growth of fusing germlings, by employing GFP and mCherry tagged protein variants (Figure 7). While the majority of the protein aggregates that formed at the plasma membrane of communicating tips contained both kinases, we frequently observed spots containing only one of the two proteins. Together, these data indicate that MEK2 and MAK2 functionally interact at the plasma membrane of fusion tips, but that they appear to be independently recruited and/or released from these kinase complexes.

NRC1-GFP and HYM1-GFP could barely be detected in germlings carrying constructs controlled by the native or the *ccg-1* promoters. Expression of a functional *nrc-1-gfp* construct under control of the *tef-1* promoter [50] resulted in a weak, but detectable GFP signal in young germlings (note that in germlings P*ccg-1* is less active than P*tef-1*). In rare cases, we observed oscillatory recruitment of the NRC1-GFP fusion protein to fusion tips, suggesting a similar subcellular dynamics of all three kinases of the MAK2 pathway (Figure 6, Figure S5). Expression of a *ptef-1-hym1-gfp* construct resulted in detectable GFP signals, but plasma membrane recruitment and dynamics of HYM1-GFP in tropic growing cell tips could not be unambiguously determined. However, after germlings had established physical contact, HYM1 and NRC1 both clearly accumulated at the future fusion site, similar to MAK2 and MEK2, indicating that the three kinases as well as HYM1 share their highly dynamic subcellular localization during the process of germling fusion. In contrast, the NDR kinase COT1 stably localized to both tips of interacting germ tubes, which likely represents its general function in hyphal polarity establishment and maintenance (Figure 6).

## Discussion

Establishing cell polarity and maintaining cellular asymmetry are essential properties that govern morphogenesis and the development of uni- and multicellular organisms. These processes require the sensing of subtle intra- or extracellular signals and the transduction of this information to various cellular outputs via intricate signaling networks. An emerging theme is that signaling cascades form molecular assemblies within cells. Their spatial organization is ensured by scaffolding proteins, which allow the compartmentalization of signaling pathways.

The findings reported in this study allow three significant conclusions. First, we show that COT1, POD6 and HYM1 physically interact, and the interaction of both kinases is dependent on HYM1. Moreover, HYM1 regulates the activity of COT1. Thus, we propose that HYM1 functions as scaffold of the COT1 NDR kinase pathway in *N. crassa* to bridge the two kinases of the MOR pathway. Intriguingly, and in contrast to the situation in unicellular fungi, Δ*hym-1* mutants do not develop the polarity defects characteristic for MOR pathway mutants in *N. crassa*. One possible interpretation of these data may be that the presence of HYM1 in the COT1 pathway is an evolutionary relic and not physiologically relevant. However, we consider this hypothesis unlikely. We base this conclusion on the findings that the deletion of *hym-1* resulted in COT1 activity that is reduced to a similar extent as described in Δ*pod-6* cells and that POD6-dependent Thr589 phosphorylation of COT1 is required for full activation of COT1 [39]. Moreover, Thr589 phosphorylation of COT1 is required for the proper localization of the NDR kinase [39]. The fact that the localization of COT1 in Δ*hym-1* is normal may explain a phenotype that does not fully resemble Δ*pod-6*. Intriguingly, we found that also mutants in the uncharacterized *N. crassa* components SOG2/NCU03360 and TAO3/NCU09740 do not display the typical hyperbranching defects characteristic for the central MOR elements *cot-1*, *pod-6* and *mob-2a/b*, but deletion strains have almost wild type characteristics (unpublished data). Thus, we speculate that overlapping functions between the two scaffolds HYM1 and TAO3 (and potentially with SOG2 - a fungal-specific protein with unknown function) may blur more drastic defects of deleting in *hym-1* on COT1 signaling. This hypothesis is consistent with the fact that HYM1 is a functional component of the MOR pathway in all ascomycete and basidiomycete fungi analyzed to date, [26], [27], [43], [44], and it would be surprising, if *N. crassa* HYM1 would represent an exception.

Second, the co-precipitation of HYM1 with NRC1, MEK2 and MAK2, and the expression of constitutive-active NRC1 and MEK2 versions and of a MEK2-NRC1 fusion protein clearly demonstrate that HYM1 is critical for the organization of the MAK2 pathway. We also show that HYM1 co-localizes with MAK2 at the hyphal apex and with all three MAK2 pathway kinases at septa and the contact point of communicating fusion cells. However, our yeast two-hybrid data also suggest that HYM1 does not physically interact with any of the three kinases of the MAK2 cascade. Because animal MO25 functions as master regulator for multiple STE20-like kinases [32], we speculate that additional kinase(s) of this group may bridge HYM1 with the three kinases of the MAK2 module as part of a functional HYM1-kinase complex that organizes the MAK2 cascade. This hypothesis is supported by the physical interaction of HYM1 and NRC1 with the *N. crassa* PAK kinase STE20, but not the related PAK kinase CLA4 in our yeast two-hybrid experiments. Interestingly, Δ*ste-20* is fusion competent and has only mild growth defects (data not shown), indicating that additional components must be part of the signal receiving machinery of the self-fusion pathway. Furthermore, our data indicate that self-signaling during germling fusion involves specific requirements for the regulation of the MAK2 module in addition to a general function of MAK2 in hyphal morphogenesis. Constitutive hyperactivation of NRC1 and MEK2 rescues the vegetative growth defects of MAK2 pathway mutants, but not their fusion defects. Together with our observation on the subcellular localization of MAK2 and its upstream kinases, this indicates that self-signaling, but not continuous hyphal growth requires a regulated on-off switch of the MAP kinase module.

Third, the individual components of the MAK2 kinase cascade display distinct localization behaviors. During self-communication and germling fusion, MEK2 and MAK2 show similar oscillating localization to the plasma membrane of interacting tips, but aggregates containing only one of the two kinases were frequently detected. The signal of the upstream kinase NRC1 was very weak and often close to the detection limit. Nevertheless, it appears to underlay a similar subcellular dynamics. This indicates that membrane-bound signaling complexes do not necessarily contain equimolar amounts of the three kinases required for self-communication, as also described for the homologous kinases of the yeast mating pathway [51]. Interestingly, MAK2 localizes in the cytosol and the nucleus, while MEK2 and NRC1 are exclusively cytosolic proteins. This suggests that only the MAP kinase shuttles between the two compartments. Moreover, MAK2 accumulates in the nucleus in Δ*hym-1* cells. This may indicate that HYM1 promotes cytosolic sequestration of MAK2. Alternatively, MAK2 in Δ*hym-1* cells is inactive and may therefore accumulate in the nucleus. During vegetative growth of mature hyphae MAK2 accumulates in the *Spitzenkörper* of the hyphal apex, while MEK2 and NRC1 are not enriched at the cell tips of hyphae. Nevertheless, all three kinases localize to the forming septum and around the mature septal pore. We have no explanation for the septum association of the MAK2 cascade, but an increasing array of signaling machinery is found to be associated with the septal pore in various filamentous fungi. Thus, this and other studies identify the septal pore as a potential signaling hub within the fungal cell (summarized in [42], [52]). The different localization behaviors of the three MAP kinase components are also reflected by different protein abundances of the three kinases. MAK2 is expressed at ca. 10-fold higher level than MEK2 and NRC1 when the three proteins are expressed from their native promoters. The expression level of HYM1 is comparable to those of NRC1 and MEK2. The relative protein abundance might thus reflect the hierarchical order within the kinase cascade.

In summary, we conclude that HYM1 functions as scaffold of the COT1 NDR kinase complex and as essential regulator of the MAK2 MAP kinase cascade. We have previously identified a complex and interdependent genetic relationships between *cot-1* and *mak-2* mutants [12]. Thus, we propose that this dual use of a common regulator in the two pathways may promote the coordination of intercellular communication, tropic tip growth and cell polarity. Further analysis is required to thoroughly test this intriguing hypothesis and to unravel this signaling network, which is essential for efficient fungal growth and adaptation.

## Materials and Methods

### Strains, media, and growth conditions

Strains used in this study are listed in Table S1 (see also [53]). General genetic procedures and media used in the handling of *N. crassa* are available through the Fungal Genetic Stock Center (www.fgsc.net).

### Two-hybrid plasmids and methods

The Matchmaker Two-Hybrid system 3 (Clontech, USA) was used according to the manufacturer's instructions. cDNA of genes of interest for two hybrid tests was amplified with primers listed in Table S2 spanning the coding region from start to stop codon as annotated by the *N. crassa* database (http://www.broadinstitute.org/annotation/genome/neurospora/MultiHome.html) and cloned either into the pGADT7 vector containing the GAL4 activation domain or into pGBKT7 containing the DNA-binding domain. Fusion proteins were (co-) expressed in *S. cerevisiae* AH109 and potential interactions determined by growth tests on SD medium lacking the amino acids adenine, histidine, leucine and tryptophane.

### Plasmid construction and fungal expression of tagged proteins

Modification of the endogenous *cot-1* and *hym-1* loci to allow expression of C-terminal GFP-tagged fusion proteins was achieved by PCR-amplification of the ORFs of both genes and 1 kb fragments of their 3′UTRs using genomic DNA with primers that incorporated *Xho*I and *Sac*I*/BamH*I sites, respectively, at the ends. The fragments were cloned into the vector pGFP::hph::loxP [54] and transformed in Δ*mus-52::barR;his-3* to ensure homologous recombination with the endogenous *cot-1* and *hym-1* loci. Transformants were backcrossed with wild type to remove the Δ*mus-52::barR* mutation and the correct genotype was confirmed by Southern analysis.

To obtain strains for the subcellular localization of MEK2 and NRC1, plasmids containing either *gfp-* or *mCherry*-fusion constructs were built based on the described plasmid pMF272 [55]. The ORFs of *mek-2* and *nrc-1* were amplified by PCR as annotated by the *N. crassa* database (http://www.broadinstitute.org/annotation/genome/neurospora/MultiHome.html) using primers listed in Table S2 and introduced via *Xba*I/*Pac*I or *BamH*I/*Pac*I, respectively, into pMF272. Constructs containing *mek-2* and *nrc-1* with the native promoters were obtained by using a forward primer binding 1 kb upstream of the ORF and the reverse primers described for the *ccg-1* constructs, as listed in Table S2, and integrated into pMF272 using *Not*I and *Pac*I, thereby replacing the *ccg-1* promoter. Plasmids containing the *tef-1* promoter were constructed by amplifying the 0.9 kb promoter region from the described plasmid pAB621 [50] using primers listed in Table S2 containing a T to A replacement at position 38, thereby destroying a present *Xba*I restriction site. The fragment was inserted into pMF272 using *Not*I and *Xba*I, replacing the *ccg-1*-promoter. mCherry constructs were obtained by replacing sgfp by mCherry, using the restriction sites *Pac*I and *EcoR*I. The final plasmids were transformed into *his-3* and *his-3;Δmek-2* or *his-3;Δnrc-1*, respectively, and the transformants were selected on media lacking histidine. For co-localization experiments plasmids were additionally transformed into *his-3;nic-3* and *his-3;trp-1* and selected on media supplemented with nicotinic acid or tryptophane.

To allow expression of myc/flag/HA-tagged fusion proteins from the *his-3* locus, the candidate ORFs were amplified with primers listed in Table S2 spanning the coding region from start to stop codon as annotated by the *N. crassa* database and cloned into the vectors pFLAGN1, pHAN1 or pCCG1-3×MYC [54], [56] to allow expression of N-terminally tagged flag-NRC1, flag-MEK2, HA-MAK2 and myc-HYM1. Constitutive-active versions of flag-NRC1 and flag-MEK2 were generated by site-directed mutagenesis according to the manufacturer's instructions (Stratagene, USA). For generation of the flag-MEK2-NRC1 fusion construct, the ORF of *mek-2* was amplified by PCR and introduced via *BamH*I into vector pFLAGN1-NRC1. The final plasmids were transformed into *his-3*, *nic-3;his-3* or *trp-1;his-3*, respectively, and were selected for complementation of the *his-3* auxotrophy. Immunoprecipitation was performed with cell extracts from fused, heterokaryotic strains that were selected by their ability to grow on minimal media lacking supplements [37]. All fusion constructs were also expressed at the *his-3* locus of the respective deletion mutant and tested for full complementation of the deletion mutant defects.

### Microscopy

Low magnification documentation of fungal hyphae or colonies was performed as described [36] using an SZX16 stereomicroscope, equipped with a Colorview III camera and Cell^D^ imaging software (Olympus). Images were further processed using Photoshop CS2 (Adobe). Fluorescence microscopy was performed as described [57], [58]. An inverted Axio Observer. Z1 (Zeiss) microscope equipped with a QuantEM 512SC camera (Photometrics) and the slidebook 5.0 software (Intelligent Imaging Innovations), or an Zeiss Axiophot 2 equipped with a pco pixelfly camera and a modified version of 4D microscopy software [59], programmed by Christian Hennig and Ralf Schnabel, were used for image acquisition. Images were deconvolved using SVI Huygens Essential software and further processed using ImageJ.

### Protein methods

Liquid *N. crassa* cultures were grown at room temperature, harvested gently by filtration using a Büchner funnel and ground in liquid nitrogen. Immunoprecipitation and COT1 kinase activity assays were performed as described [37], [39]. Monoclonal mouse α-HA (clone HA-7, Sigma Aldrich, Germany), monoclonal mouse α-FLAG M2 antibody (Sigma-Aldrich, Germany), monoclonal mouse α-myc (9E10, Santa Cruz, USA), monoclonal mouse α-GFP (Invitrogen GmbH, Germany) antibodies and GFP-Trap beads (ChromoTek, Germany) were used in this study.

Protein extraction for the analysis of the MAK2 phosphorylation status was performed as described in [47] with minor modifications. Briefly, the frozen mycelial powder was incubated in 95% ethanol at -20°C for ≥12 h, the supernatant removed after centrifugation and the pellet vacuum-dried in a SpeedVac concentrator (Thermo Fisher Scientific, USA). Extraction buffer (100 mM Tris pH7.0, 1% (w/v) SDS; supplemented with 5 mM NaF, 1 mM PMSF, 1 mM Na_3_VO_4_, 25 mM β-glycerophosphate, 2 mM benzamidine, 2 ng/µl pepstatin A, 10 ng/µl aprotinin, 10 ng/µl leupeptin) was added, the samples mixed and incubated at 80°C for 5 min and the supernatant collected after centrifugation. After a second round of extraction, the supernatants pooled, subjected to another centrifugation step, and the protein concentration determined using a Nanodrop spectrophotometer (ND-1000, Peqlab, Germany). Sample volumes corresponding to 75 µg total protein per lane were subjected to SDS polyacrylamide gel electrophoresis and subsequent Western blotting using polyclonal rabbit α-Phospho-p42/44 MAPK (Cell Signaling Technology, Inc., USA) and goat α-rabbit IgG-HRP (Santa Cruz, USA) as primary and secondary antibodies, respectively. For quantification of MAK1 and MAK2 phosphorylation levels, exposed films were scanned at a resolution of 600 dpi and densitometry was performed on the resulting tiff-files employing the AIDA Image Analyzer (version 4.22; raytest Isotopenmessgeräte, Germany) in transmission mode. Intensity values [arbitrary units] measured within a region of interest of fixed size containing the MAK1 or MAK2 protein bands were corrected by subtraction of local background, normalized to the protein amount loaded and used for further evaluation.

## Supporting Information

Figure S1 Expression analysis of the used GFP fusion constructs under the control of the *ccg-1* and their native promoters. (A) Anti-GFP Western blot of normalized cell extracts of strains expressing GFP fusion proteins under the control of the indicated promoters. (B) Quantification of the relative expression levels of the indicated proteins under the control of their native promoters. Protein levels are normalized to MAK2 abundance (n = 3). (C) Quantification of the relative expression levels of the indicated proteins under the control of the indicated promoters. Protein levels are normalized to *ccg-1* promoter driven expression (n = 3).
(PSD)

Figure S2 HYM1 has a weaker binding affinity to components of the MAK2 pathway than the kinases among each other. Co-immunoprecipitation experiments between HYM1 and MEK2 (A) and between MAK2 and MEK2 (B), in which the bound precipitates were either washed twice with lysing buffer (left panels) or three times in lysing buffer supplemented with 300 mM NaCl (right panels). Note that the interaction between HYM1 and MEK2, but not between MAK2 and MEK2 is abolished under the high salt washing conditions.
(PSD)

Figure S3 Phenotypic characteristics of constitutive hyperactive NRC1 and MEK2 variants. Phenotypic characterization of the strains described in Table 1 regarding macroscopic morphology and conidiation pattern (left panel), germling fusion (upper right panel), and protoperithecia formation (lower right panel).
(PSD)

Figure S4 The promoter strength does not influence the apical localization of MAK2-GFP. Localization of MAK2-GFP expressed under the control of the *ccg-1* promoter (A) and its native promoter (B) resulted in comparable localization patterns at the hyphal tip and the septum.
(PSD)

Figure S5 Oscillating recruitment of NRC1-GFP to the tips of interacting germlings. NRC1-GFP is expressed under the control of the *tef-1* promoter. Between the two time points shown, tip localization has switched between the two germlings. Upper images: DIC, lower images: GFP Fluorescence.
(PSD)

Table S1 *Neurospora crassa* strains used in this study.
(DOC)

Table S2 Primers used in this study. Restriction enzyme recognition sites are indicated in bold, lower case letters and mismatched nucleotides for insertion of mutations are depicted in italic, lower case letters.
(DOC)

Video S1 Time-course of COT1-GFP localization during septum formation. COT1-GFP formed cortical rings at incipient septation sites that constricted during septum formation and accumulated around the septal pore of the completed septum (a) GFP channel; (b) FM4-64 channel; (c) merged. The plasma membrane was stained with FM4-64. Images were captured at 15 sec. intervals.
(AVI)

Video S2 Time-course of HYM1-GFP localization during septum formation. HYM1-GFP formed cortical rings at incipient septation sites that constricted during septum formation and accumulated around the septal pore of the completed septum (a) GFP channel; (b) FM4-64 channel; (c) merged. The plasma membrane was stained with FM4-64. Images were captured at 15 sec. intervals.
(AVI)

Video S3 Time-course of MAK2-GFP localization during septum formation. MAK2-GFP formed cortical rings at incipient septation sites that constricted during septum formation and accumulated around the septal pore of the completed septum (a) GFP channel; (b) FM4-64 channel; (c) merged. The plasma membrane was stained with FM4-64. Images were captured at 15 sec. intervals.
(AVI)

Video S4 Time-course of MEK2-GFP localization during septum formation. MEK2-GFP formed cortical rings at incipient septation sites that constricted during septum formation and accumulated around the septal pore of the completed septum (a) GFP channel; (b) FM4-64 channel; (c) merged. The plasma membrane was stained with FM4-64. Images were captured at 15 sec. intervals.
(AVI)

Video S5 Time-course of NRC1-GFP localization during septum formation. NRC1-GFP formed cortical rings at incipient septation sites that constricted during septum formation and accumulated around the septal pore of the completed septum (a) GFP channel; (b) FM4-64 channel; (c) merged. The plasma membrane was stained with FM4-64. Images were captured at 15 sec. intervals.
(AVI)
