# Supplementary material for: The NDR Kinase Scaffold HYM1/MO25 Is Essential for MAK2 MAP Kinase Signaling in Neurospora crassa
Source: PLoS Genet. 2012 Sep 20;8(9):e1002950. doi: 10.1371/journal.pgen.1002950 (PMC3447951; doi:10.1371/journal.pgen.1002950)
Supplement: Table S1 — Neurospora crassa strains used in this study. (DOC) [file pgen.1002950.s006.doc]

**Suppl. Table 1.** *Neurospora crassa* strains used in this study

| Strains | Genotype | Source |
| --- | --- | --- |
| *wild type 74* | *OR231 Mat A* | FGSC #987 |
| *wild type ORS* | *SL6 Mat a* | FGSC #4200 |
| *his-3 A* | *his-3 Mat A* | FGSC #6103 |
| *his-3 a* | *his-3 Mat a* | FGSC #718 |
| *trp-1;his-3* | *trp-1- his-3-* |  |
| *nic-3;his-3* | *nic-3- his-3-* |  |
| *∆mus52::bar;his-3* | *mus52∆::barR his-3-* | FGSC #9720 |
| *∆hym-1* | *hph::hym-1∆* | FGSC #13043 |
| *∆nrc-1* | *hph::nrc-1∆* | FGSC #18162 |
| *∆ste-7* | *hph::ste-7∆* | FGSC #11481 |
| *∆mak-2*  *∆mak-1* | *hph::mak-2∆*  *hph::mak-1∆* | FGSC #11482  FGSC #11318 |
| *myc-cot-1;his-3*  *myc-cot-1;his-3;∆hym-1*  *myc-cot-1;Pccg-1-hym-1-gfp;∆hym-1* | *Pcot-1-myc-cot-1 his-3-*  *Pcot-1-myc-cot-1 his-3- hph::hym-1∆*  *Pcot-1-myc-cot-1 Pccg-1-hym-1-sgfp::his-3 hph::hym-1∆* | This study  This study |
| *myc-cot-1* | *Pcot-1-myc-cot-1* |  |
| *HA-pod-6* | *Ppod-6-HA-pod-6* | (Maerz et al., 2012) |
| *HA-pod-6;myc-cot-1;his-3* | *Pcot-1-myc-cot-1 Ppod-6-HA-pod6 his-3-* | This study |
| *HA-pod-6;myc-cot-1;∆hym-1;his-3* | *Pcot-1-myc-cot-1 Ppod-6-HA-pod-6 hph::hym-1∆ his-3-* | This study |
| *Phym-1-hym-1-gfp* | *Phym-1-hym-1-sgfp::hph* | This study |
| *Pccg-1-hym-1-gfp*  *Ptef-1-hym-1-gfp* | *Pccg-1-hym-1-sgfp::his-3*  *Ptef-1-hym-1-sgfp::his-3* | This study  This study |
| *Pccg-1-hym-1-gfp trp-1* | *Pccg-1-hym-1-sgfp::his-3 trp-1* | This study |
| *cot-1-gfp* | *Pcot-1-cot-1-sgfp::hph* | (Maerz et al. 2012) |
| *cot-1-gfp;∆hym-1* | *Pcot-1-cot-1-sgfp::hph::hym-1∆* | This study |
| *AF-M512* | *Pccg-1-mak2-sgfp::his-3 hph::mak-2∆* |  |
| *P611-3* | *Pmak-2-mak-2-sgfp::his-3 hph::mak-2∆* |  |
| *Pccg-1-mak-2-gfp, trp-1* | *Pccg-1-mak-2-sgfp::his trp-1-* | This study |
| *Pccg-1-mak-2-cherry, nic-3* | *Pccg-1-mak-2-cherry::his nic-3-* | This study |
| *Pccg-1-mak-2-gfp;∆hym-1* | *Pccg-1-mak2-sgfp:: hph::hym-1∆* | This study |
| *Pccg-1-mek-2-gfp* | *Pccg-1-mek-2-sgfp::his-3 hph::mek-2∆* | This study |
| *Ptef-1-mek-2-gfp* | *Ptef-1-mek-2-sgfp::his-3* | This study |
| *Pccg-1-mek-2-cherry, nic-3* | *Pccg-1-mek-2-mcherry::his-3 nic-3-* | This study |
| *Pccg-1-mek-2-gfp;∆hym-1* | *Pccg-1-mek-2-sgfp:: hph::hym-1∆* | This study |
| *Pccg-1-nrc1-gfp* | *Pccg-1-nrc1-sgfp::his-3 hph::nrc-1∆* | This study |
| *Ptef-1-nrc-1-gfp* | *Ptef-1-nrc-1-sgfp::his-3* | This study |
| *Pccg-1-nrc1-gfp;∆hym-1* | *Pccg-1-nrc1-sgfp::his-3 hph::hym-1∆* | This study |
| *Pccg-1-myc-hym-1;trp-1* | *Pccg-1-3xmyc-hym-1::his-3 trp-1-* | This study |
| *Pccg-1-myc-hym-1;nic-3*  *Pccg-1-hym-1-gfp;trp-1* | *Pccg-1-3xmyc-hym-1::his-3 nic-3-*  *Pccg-1-hym-1-sgfp::his-3 trp-1-* | This study  This study |
| *Pccg-1-HA-mak-2;trp-1* | *Pccg-1-3xHA-mak-2::his-3 trp-1-* | This study |
| *Pccg-1-HA-mak-2;nic-3* | *Pccg-1-3xHA-mak-2::his-3 nic-3-* | This study |
| *flag-nrc-1;trp-1* | *Pccg-1-3xflag-nrc-1::his-3 trp-1-* | This study |
| *flag-nrc-1;nic-3* | *Pccg-1-3xflag-nrc-1::his-3 nic-3-* | This study |
| *flag-nrc-1(P488S)* | *Pccg-1-3xflag-nrc-1(P488S)::his-3* | This study |
| *flag-nrc-1;Δhym-1* | *Pccg-1-3xflag-nrc-1::his-3 hph::hym-1∆* | This study |
| *flag-nrc-1(P488S);Δhym-1* | *Pccg-1-3xflag-nrc-1(P488S)::his-3 hph::hym-1∆* | This study |
| *flag-nrc-1;Δnrc-1* | *Pccg-1-3xflag-nrc-1::his-3 hph::nrc-1∆* | This study |
| *flag-nrc-1(P488S);Δnrc-1* | *Pccg-1-3xflag-nrc-1(P488S)::his-3 hph::nrc-1∆* | This study |
| *flag-nrc-1(P488S);Δste-7* | *Pccg-1-3xflag-nrc-1(P488S)::his-3 hph::mek-2∆* | This study |
| *flag-mek-2;nic-3* | *Pccg-1-3xflag-mek-2::his-3 nic-3-* | This study |
| *flag-mek-2;trp-1* | *Pccg-1-3xflag-mek-2::his-3 trp-1-* | This study |
| *flag-mek-2(S212D;T216D)* | *Pccg-1-3xflag-mek-2(S212D;T216D)::his-3* | This study |
| *flag-mek-2;*Δ*hym-1* | *Pccg-1-3xflag-mek-2::his-3 hph::hym-1∆* | This study |
| *flag-mek-2(S212D;T216D);Δhym-1* | *Pccg-1-3xflag-mek-2(S212D;T216D)::his-3 hph::hym-1∆* | This study |
| *flag-mek-2;*Δ*mek-2* | *Pccg-1-3xflag-mek-2::his-3 hph::mek-2∆* | This study |
| *flag-mek-2(S212D;T216D);*Δ*mek-2* | *Pccg-1-3xflag-mek-2(S212D;T216D)::his-3 hph::mek-2∆* | This study |
| *flag-mek-2-nrc-1* | *Pccg-1-3xflag-MEK-2-nrc-1* | This study |
| *flag-mek-2-nrc-1;*Δ*hym-1* | *Pccg-1-3xflag-MEK-2-nrc-1 hph::hym-1∆* | This study |
| *h1-rfp* | *Pccg-1-rfp-h1::his-3* | M. Riquelme, Mexico |
