# Supplementary material for: The NDR Kinase Scaffold HYM1/MO25 Is Essential for MAK2 MAP Kinase Signaling in Neurospora crassa
Source: PLoS Genet. 2012 Sep 20;8(9):e1002950. doi: 10.1371/journal.pgen.1002950 (PMC3447951; doi:10.1371/journal.pgen.1002950)
Supplement: Table S2 — Primers used in this study. Restriction enzyme recognition sites are indicated in bold, lower case letters and mismatched nucleotides for insertion of mutations are depicted in italic, lower case letters. (DOC) [file pgen.1002950.s007.doc]

**Suppl. Table 2.** Primers used in this study

| **Name** | **Sequence 5‘- 3‘** |
| --- | --- |
| Modifications of endogenous loci: |  |
| DJ_Cot_Start_XhoI5 | **ctc gag** ATG GAC AAC ACC AAC CGC |
| DJ_Cot_Stopp_XhoI3 | **ctc gag** CTC GGA ATG TGT TGT CGA AAC |
| ADendoGFPhym1-XhoI5 | **ctc gag** ATG TCA TTC CTC TTT GG |
| ADendoGFPhym1-XhoI3 | **ctc gag** CCC GCT GCG GCA C |
| DJ_Cot_3UTR_SacI5 | **gag ctc** TTG CGT TAC GTC TTC TCG CTG |
| DJ_Cot_3UTR_SacI3 | **gag ctc** GTT ATA TTA GAA GAA AGT ACT T |
| ADendoGFPhym1-XhoI5 | **ctc gag** ATG TCA TTC CTC TTT GG |
| ADendoGFPhym1-XhoI3 | **ctc gag** CCC GCT GCG GCA C |
| ADendoGFPhym1-BamHI-3UTR5 | **gga tcc** GGT AAC TAA TTG GTC TG |
| ADendoGFPhym1-BamHI-3UTR3 | **gga tcc** TTT TTT CCT TCT TTT CTC |
| 3xFLAG-tag constructs: |  |
| ADFLAGnrc1-BamHI5 | **gga tcc** ATG GCC ATG CTG GC |
| ADFLAGnrc1-EcoRI3 | **gaa ttc** CTA TGT CCC GGG CAC |
| ADste7_BamHI5 | **gga tcc** ATG GCC GAC CCA TTT |
| ADste7_PacI3 | **tta att aa**T CAA AAT CGG CCA GTT G |
| ADFLAGste7-BamHI3 | **gga tcc** TCC GCC AAA TCG GCC AG |
| 3xHA-tag constructs: |  |
| ADmak2_BamHI5 | **gga tcc** ATG AGC AGC GCA CAA A |
| ADmak2_PacI3 | **tta att aa**T CAC CTC ATA ATC TCC TG |
| 3xmyc-tag constructs: |  |
| ADMYChym1-SgsI5 | **ggc gcg cc**T ATG TCA TTC CTC TTT GGG AGA G |
| ADMYChym1-SpeI3 | **act agt** TTA CCG CTG CGG CAC C |
| yeast two hybrid constructs: |  |
| ADmak2_EcoRI5 | CAG T**ga att c**AT GAG CAG CGC ACA AAG AGG C |
| ADmak2_BamHI3 | GAT **gga tcc** TCA CCT CAT AAT CTC CTG GTA G |
| ADste7_EcoRI5 | CAG **tga att c**AT GGC CGA CCC ATT TGC CC |
| ADste7_BamHI3 | GAT **gga tcc** TCA AAA TCG GCC AGT TGG AGG |
| ADnrc1_EcoRI5 | CAG T**ga att** CAT GGC CAT GCT GGC ATC CAA G |
| ADnrc1_BamHI3 | GAT **gga tcc** CTA TGT CCC GGG CAC AGG CG |
| ADste20_GAD_SmaI5 | CCA **ccc ggg** TAT GGA CGG CCA GCT CTC CC |
| ADste20_GAD_XhoI3 | CAG **ctc gag** CTA CTG CCC TTT ACG GGC C |
| ADcla4_GAD_NdeI5 | CT**c ata tg**G CGC AAA ATG GCA ACA TAT ACT |
| ADcla4_GAD_EcoRI3 | GTG **gaa ttc** TTA TTT CGC CGA CCT CTT GAA G |
| hym1Eco-5 | G**ga att c**AT GTC ATT CCT CTT TGG GAG AG |
| hym1Bam-3 | CG**g gat cc**T TAC CGC TGC GGC ACC ACG |
| COT1 Eco-5 | G**ga att c**AT GGA CAA CAC CAA CCG CC |
| COT1 Bam-5 | CG**g gat cc**T TAT CGG AAG TTG TTG TCG AAA C |
| POD6 Nde-5 | GAT CAG **cat atg** GCG ACC CTA TCG GTA TAC |
| POD6 Eco-3 | G**ga att c**CT ACC TCC CTC AGA CAC TCG TG |
| constitutive-active and  STE7-NRC1 fusion plasmids: |  |
| ADnrc1MutP_F | cag ttc ggt ggg ctg aga *tcg* cct agc gag ctt atc gcc |
| ADnrc1MutP_R | ggc gat aag ctc gct agg *cga* tct cag ccc acc gaa ctg |
| ADste7_MutP_F | GAG AGC TAG TTA AC*g at*G TCG CCG AC*g ac*T TTG TGG GTA CAT C |
| ADste7_MutP_R | GAT GTA CCC ACA AA*g tc*G TCG GCG AC*a tc*G TTA ACT AGC TCT C |
| ADFUSste7-BamHI5 | **gga tcc** ATG GCC GAC CCA TTT |
| ADFLAGste7-BamHI3 | **gga tcc** TCC GCC AAA TCG GCC AG |
| GFP-fusion plasmids: |  |
| Nrc1 BamHI | GTCGCTGGAT**ccatgg**CCATGCTGGCATCCAAGT |
| NRC-1-FII | ATTAATA**gcggccgC**TTGTCTCTCTCTCTCCTCCAGCAGC |
| Nrc1 PacI | AGACTC**ttaattaa**TGTCCCGGGCACAGGCGTCAAGA |
| Ste7 XbaI | GCTTAAACAAACA**tctaga**ATGGCCGACCCATTT |
| STE-7-FII | ATTAATA**gcggccgc**TCCTAGTCTTCAGTTCCTTACCG |
| Ste7 PacI | CTCAAC**ttaattaa**AAATCGGCCAGTTGGAGGCTGCA |
| TEF-1 mutXba | GGTG**gcggccgc**GATATCCCGTGACCACTGAACTACACTAGTC*a*AGAGTGAAGCTTGTGG |
| TEF-1 R | AATTCTAGATAACCCGGGGATCCGATATC |

Restriction enzyme recognition sites are indicated in bold, lower case letters and mismatched nucleotides for insertion of mutations are depicted in italic, lower case letters.
